# Micromechanics-based constitutive relations for post-localization analysis

**DOI:** 10.1016/j.mex.2018.10.018

**Published:** 2018-10-24

**Authors:** Mohammad E. Torki, A. Amine Benzerga

**Affiliations:** a Department of Aerospace Engineering, Texas A&M University, College Station, TX, 77843, United States; b TEES Center for intelligent Multifunctional Materials and Structures (CiMMS), College Station, TX, 77843, United States; c Department of Materials Science & Engineering, Texas A&M University, College Station, TX, 77843, United States

**Keywords:** Post-localization analysis in shear, Ductility, Void growth, Void coalescence, Lode angle, Triaxiality

## Abstract

Micromechanics-based constitutive relations for post-localization analysis are obtained, to be used in a multi-surface representation of porous metal plasticity. Each yield surface involves a number of internal parameters. Hence, the constitutive relations must be closed with evolution equations for the internal parameters. The latter are essential to describing the gradual loss of load-bearing capacity under shear-dominated loading. We also briefly discuss potential void closure due to void rotation and elongation in shear and show additional details regarding the simulations reported in a recent paper (A mechanism of failure in shear bands (2018) Extreme Mechanics Letters, **23**, pp. 67-71.) The method can be more broadly used in a range of ductile failure problems involving combined tension and shear loadings.

## Graphical abstract

**Figure:**
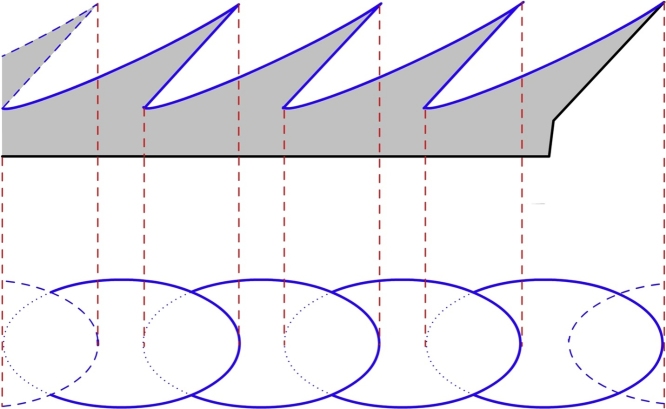

**Specifications table**

**Table:** 

**Subject Area**	*Engineering*
**More specific subject area:**	*Mechanics of solids*
**Method name:**	*Post-localization analysis in shear*
**Name and reference of original method**	*S. Kweon, B. Sagsoy, and A. A. Benzerga, Comput. Methods Appl. Mech. Eng. 310, 495 (2016).*
**Resource availability**	*If applicable, include links to resources necessary to reproduce the method (e.g. data, software, hardware, reagent)*

## Method details

Simulations of void-mediated failure in shear have recently been carried out [1]. The simulations were based on a two-surface porous material plasticity formulation. The two surfaces represent different regimes of void growth, dependent on whether plastic flow is localized at the sub-cell level or not. A complete set of constitutive relations was reported in [1], including a list of evolution equations for the internal parameters. Here, the methods involved in developing the evolution equations post-localization are presented in detail.

The natural framework to describe elastic deformation is Lagrangian. On the other hand, the natural framework to describe plastic flow is Eulerian. Formulations of elasto-plastic constitutive relations commonly adopt an additive decomposition of the total velocity gradient within an Eulerian setting [2,3]:

(1) $$L=L^{e}+L^{p}$$

so that a weak form of elasticity (hypo-elasticity) is employed for $L^{e}$. Here, we describe how we formulate $L^{p}$ in the context of a two-surface representation of dilatant plasticity. Each yield surface involves a number of internal parameters. Hence, the constitutive relations must be closed with evolution equations for these. Such equations are essential to describing the gradual loss of load bearing capacity in shear, as presented in [1]. We also briefly discuss potential void closure due to void rotation and elongation in shear. In this regard, we show some additional details regarding the simulations reported in [1].

### Two-surface formulation

Background on the two-surface formulation may be found in [4,5]; also see [6,7] for recent perspectives. The effective yield surface is the intersection of two surfaces. The first, expressed in the form $\Phi^{\text{H}}\left(\sigma ;f,w,n^{\left(3\right)}\right)=0$, represents homogeneous plastic flow, at an appropriate scale of description, and involves three internal parameters: void volume fraction, *f*, void aspect ratio, *w*, and void orientation, **n**^(3)^. It is used to describe void growth, and encompasses the famous Gurson model [8] for spherical voids under the constraint of no shape change. In our implementation, we used the model developed in [9]. The second yield surface, written as $\Phi^{\text{I}}\left(\sigma ;f,w,\lambda ,n^{\left(3\right)},n\right)=0$, corresponds to inhomogeneous plastic flow and involves two additional internal parameters: the orientation of the localized band, **n**, and the relative void spacing^1^, λ, associated with **n**. It is used to describe void coalescence, and encompasses the recently developed models of void coalescence in tension [10] and under combined tension and shear [11]. Here, the following expression is used for $\Phi^{\text{I}}$ [11]

(2) $$\Phi^{I}=\left\{\begin{array}{l} \begin{array}{ccc} {\left(\frac{\left|\sigma \right|-S(\bar{\chi },\bar{w})}{\nu (\bar{\chi })}\right)}^{2}+\frac{\tau^{2}}{(1-{\bar{\chi }}^{2}){\bar{\tau }}^{2}}-1 & \text{for} & \left|\sigma \right|\geq S \end{array}\\ \begin{array}{ccc} \frac{\tau^{2}}{(1-{\bar{\chi }}^{2}){\bar{\tau }}^{2}}-1 & \text{for} & \left|\sigma \right|<S \end{array} \end{array}\right\}$$

where

(3) $$\nu /\bar{\tau }=2-\sqrt{1+3{\bar{\chi }}^{4}}+\ln\frac{1+\sqrt{1+3{\bar{\chi }}^{4}}}{3{\bar{\chi }}^{2}}$$

(4) $$S/\bar{\tau }=\frac{{\bar{\chi }}^{3}-3\bar{\chi }+2}{3\bar{\chi }\bar{w}}$$

where the effective ligament parameter, $\bar{\chi }$, and effective void aspect ratio, $\bar{w}$, correspond to an equivalent cylindrical void with axis **n**, obtained by a volume-preserving projection of the rotating void onto the localization plane. Implicit dependence upon the void axis in (2) is through *w* and dependence upon the localization plane normal is through $\bar{\chi }$ and $\bar{w}$ as well as σ=n.σn and τ=m.σn with **m** a unit vector along the applied shear.

The two-surface formulation, also known as the hybrid model, was discussed in a recent review [7]. Since the plastic portion of the velocity gradient, $L^{p}$, is obtained by normality to the effective yield surface, the hybrid model presents the disadvantage of an ill-defined direction of plastic flow if the current loading point lies on a vertex of the yield surface. This problem arises because yield functions $\Phi^{\text{H}}$ and $\Phi^{\text{I}}$ were actually obtained independently for two elementary cells using micromechanics. To remedy this problem, a unified model has recently been developed [12]. The resulting yield surface exhibits regions of extreme curvature near the vertices of the hybrid model, but is fully smooth. This shortcoming of the hybrid model has no consequences on the results presented in [1] for a simple reason: under near simple shear, plastic flow is inhomogeneous from the outset. In other words, the current loading point is far from any vertex.

### Surrogate microstructure

**Fig. 1:**
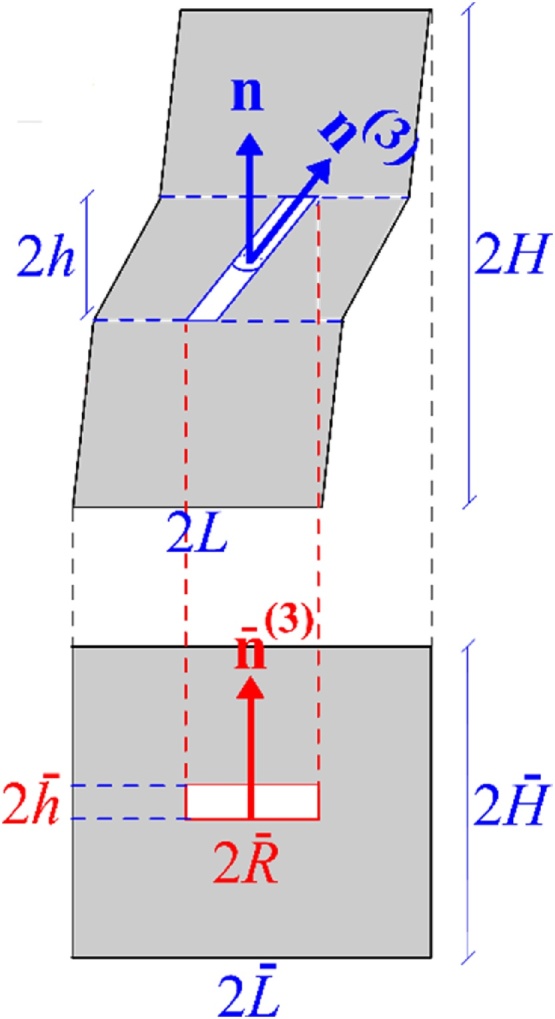
Concept of surrogate or intermediate configuration.

The concept of a surrogate microstructure involves replacing the rotating void with an upright cylinder of axis **n**, having the same volume and porosity, Fig. 1.

This identification is needed for applicability of yield function $\Phi^{\text{I}}$; see Eq. (2) above. It leads to introduction of an effective void aspect ratio, $\bar{w}=\bar{h}/\bar{R}$, and an effective ligament parameter, $\bar{\chi }=\bar{R}/\bar{L}$, related to the internal parameters of the actual microstructure through:

(5) $$\bar{w}=w{\left(wS+\frac{1}{C}\right)}^{-3}$$

and

(6) $$\bar{\chi }={\left(\frac{f\bar{\lambda }}{\bar{w}}\right)}^{\frac{1}{3}}$$

where use has been separately made of equality in void volumes and cell volumes. Here, *C* and *S* are short-hand notation for $C=n^{\left(3\right)}.n\equiv \cos\theta$ and $S=n^{\left(3\right)}.m\equiv \sin\theta$. Also, $\bar{\lambda }$ denotes the aspect ratio of the surrogate cell and is obtained from:

(7) $$\bar{\lambda }=\frac{1}{{\left(1+\gamma_{\text{mn}}\right)}^{3}}$$

where $\gamma_{\text{mn}}=2m.En$ and E=∫Ddt.

The concept of a surrogate microstructure is key to the predictions discussed in [1]. In simple shear, the void rotates “faster" than the material so that $\bar{\chi }$ would evolve, unlike the actual ligament parameter χ=R/L.

**Fig. 2:**
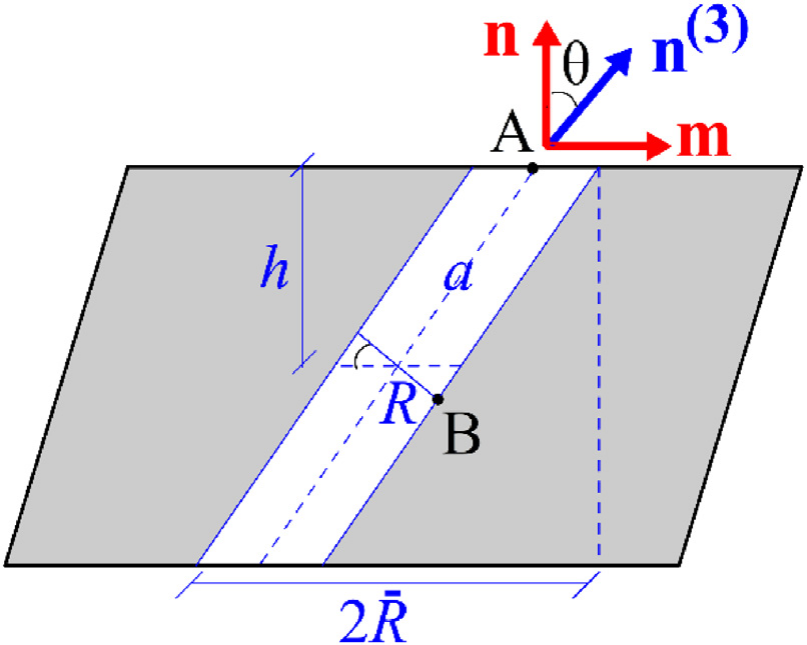
Inclined cylindrical void inside a deformed ligament under the effect of shear τ along **m** and normal stress σ along **n.**

It is worth noting that an elementary estimation of the limit load in simple shear for the inclined cylinder of Fig. 1a delivers $\tau =(1-\chi^{2})\bar{\tau }$ irrespective of the void inclination, with χ, not $\bar{\chi }$, appearing in the equation. This simple estimate is contrary to the projection-guided estimate of Eq. (2). One cannot emphasize enough, however, that the elementary estimate is obtained using Gurson’s shear field, also used in [11]. Presumably, this field becomes increasingly poor for inclined voids and large values of χ. An indication of that may be inferred from three-dimensional calculations for elongated voids reported in [13]. A qualitative theoretical argument supporting this is as follows. Let **n**^(1)^ be the unit vector perpendicular to **n**^(3)^ lying in the shearing plane, i.e. **n**-**m** plane (Fig. 2). The lateral void boundaries (having **n**^(1)^ as a normal) are traction-free, hence:

(8) $$n^{\left(1\right)}.\sigma n^{\left(1\right)}=n^{\left(3\right)}.\sigma n^{\left(1\right)}=0$$

The latter equation implies no shear plastic strain accumulation on those surfaces

(9) $$n^{\left(3\right)}.D^{p}n^{\left(1\right)}=0$$

where use has been made of the flow rule associated with the von Mises yield criterion tacitly assumed in the matrix for deriving the overall response. However, for large inclinations of the void, vectors **n**^(1)^ and **n**^(3)^ nearly coincide with **n** and **m**. It follows that the no-shearing condition above becomes

(10) $$n.D^{p}m\approx 0$$

This condition is obviously violated by Gurson's shear velocity field, which leads to a uniform deviatoric strain rate and in particular to a uniform and *non-zero* value of $n.D^{p}m$.

The above argument means that Gurson's shear field becomes increasingly inadequate in those parts of the intervoid ligament that are close to the boundary of the inclined void. Furthermore, for inclined voids, this ligament is wide (in the direction of vector **n**^(3)^) and thin (in the direction of vector **n**^(1)^), see Fig. 5a in Ref. [1]. Therefore, most of the intervoid ligament lies close to the boundary of the void, making the inadequacy of Gurson's field more pronounced.

In summary, the proposed heuristics is based on the so-justified assumption that, for an inclined cylinder, the limit load in shear would depend not only on χ but also on the void orientation relative to the cell's. This is precisely what surrogate parameter $\bar{\chi }$ captures, albeit approximately.

### Evolution equations

Differential equations describing the evolution of internal variables in $\Phi^{\text{H}}$ were part of the developments in Ref. [9]. However, those associated with $\Phi^{\text{I}}$ were not developed in Ref. [11]. They were listed in [1] as Eqs. (4), (5), (6), (7), (8), (9), (10). In particular, those pertaining to void shape and orientation, Eqs. (5), (6), (7), (8) therein, are original and thus require special attention.

Consistent with the void geometry considered in deriving $\Phi^{\text{I}}$ in [11], we consider a deformed configuration under shear deformation, Fig. 2. Only the ligament region, of height 2*h*, is shown for clarity. Recall that elastically unloaded zones lie above and below the void, as sketched in Fig. 1. For simplicity, these zones are modeled as rigid in what follows.

#### Evolution of void shape

To obtain the differential equation for void aspect ratio *w*, we first express that the top and bottom void boundaries are attached to the rigid zones. Due to symmetry, we focus on motion of the top boundary. Thus, the tangential and normal velocities of point A (Fig. 2) are given by:

(11) $$v_{1}^{\left(A\right)}=2h\frac{D_{31}}{c}=2HD_{31},\;\;\;\;\;\;\;\;v_{3}^{\left(A\right)}=h\frac{D_{33}}{c}=HD_{33}$$

where the *x*_1_ and *x*_3_ axes are identified with the directions of shear, **m**, and normal to the band, **n**, respectively. Accordingly, *D*_31_ and *D*_33_ are the relevant (non−zero) components of the plastic strain rate (superscript “p” dropped for convenience and elastic strain rates neglected). Also, c=h/H denotes the current ligament volume fraction.

Denoting the length of the inclined cylinder and its radius by 2*a* and 2*R*, respectively (see Fig. 2), the time rate of *a* is obtained at fixed void orientation θ as

(12) $$\dot{a}=v_{1}^{\left(A\right)}S+v_{3}^{\left(A\right)}C=H\left(2D_{31}S+D_{33}C\right)$$

Hence, using the identity H/a=C/c one gets

(13) $$\frac{\dot{a}}{a}=\frac{C}{c}\left(2D_{31}S+D_{33}C\right)$$

The ligament volume fraction, *c*, is given by

(14) $$c^{3}=C^{3}\frac{fw^{2}}{\lambda^{2}}$$

Next, the rate of change of *R* may be obtained (in terms of that of *a*) from plastic incompressibility of the matrix material as

(15) $$\frac{\dot{R}}{R}=\frac{1}{2}\left[{\left(\frac{L}{R}\right)}^{2}\frac{H}{a}D_{33}-\frac{\dot{a}}{a}\right]=\frac{1}{2}\left(\frac{D_{33}}{f}-\frac{\dot{a}}{a}\right)$$

Combining (13) and (15) leads to the following evolution equation for the void aspect ratio:

(16) $$\frac{\dot{w}}{w}=\frac{\dot{a}}{a}-\frac{\dot{R}}{R}=\frac{1}{2}\left(\frac{3C^{2}}{c}-\frac{1}{f}\right)n.D^{p}n+\frac{3CS}{c}m.D^{p}n$$

#### Evolution of void orientation

In general, the rate of rotation of the (immaterial) principal axes of the void may be directly obtained from the (material) rotation and strain rates of the void [14,15], denoted by **Ω**^v^ and ***D***^v^, respectively. Here, we obtain **Ω**^v^ as in [14,15] but specialized to the ligaments only. However, the contribution to the rotation rate of the axes that comes from mere void distortion (i.e. that tied to ***D***^v^) is rederived from first principles. Madou and Leblond [15] have shown that the general form initiated in [14,16] requires significant amendments due to strong nonlinear effects. They did so by introducing heuristic coefficients calibrated using a large number of finite-element based limit analyses. Here we obtain simpler, parameter-free and probably more accurate equations by considering the constrained kinematics pertaining to post-localization. Namely, this involves plastic incompressibility of the intervoid ligament and the fact that the top and bottom boundaries of the void move rigidly with the above and bottom material layers. Such equations are obviously valid only for post-localization. Thus, the rate of change of the void axis is given by Eq. (6) of [1], rewritten here for completeness:

(17) $${\dot{n}}^{\left(3\right)}=\omega n^{\left(3\right)},\omega =\Omega^{v}+\Omega^{l}$$

where the rotation tensor **ω** accounts for the void spin, **Ω**^v^, which is determined as in [14]. It is related to the continuum spin tensor **Ω** via the following

(18) $$\Omega^{v}=\Omega -\frac{1}{c}\mathbb{C}:D^{p}$$

where ℂ is the fourth order spin concentration tensor given by

(19) $$\mathbb{C}=-\left(1-f\right)\mathbb{P}:A,\;\;\;\;\;\;A={\left[I-\left(1-f\right)S\right]}^{-1}$$

with A the strain concentration tensor and ℙ and S the Eshelby tensors [17] for a spheroidal inclusion of zero stiffness in an incompressible linear viscous matrix. Note that a 1/c term appears in (18) to represent the plastic rate of deformation inside the ligament.

Also, $\Omega^{l}$ in (17) is an additional contribution to the effective void rotation that comes from mere distortion of void boundaries under the combined effect of tension and shear. With reference to (14), the time rate of *c* reads

(20) $$3\frac{\dot{c}}{c}=3\frac{\dot{C}}{C}+\frac{\dot{f}}{f}+2\left(\frac{\dot{w}}{w}-\frac{\dot{\lambda }}{\lambda }\right)$$

The rates of internal parameters entering the right-hand side of this equation are all known, except the void orientation, which enters through *C*. The left-hand side can be determined by neglecting the volume change of the elastically unloaded zones. Thus,

(21) $$\frac{\dot{c}}{c}=\frac{\dot{h}}{h}-\frac{\dot{H}}{H}=\frac{D_{33}}{c}-D_{33}=\frac{1-c}{c}n.D^{p}n$$

Also,

(22) $$\frac{\dot{\lambda }}{\lambda }=\frac{\dot{H}}{H}-\frac{\dot{L}}{L}=n.D^{p}n-\frac{m.D^{p}m+p.D^{p}p}{2}$$

where p=n×m completes the triad of local base vectors. Therefore, Eq. (20) may be used to determine $\dot{C}/C$. Furthermore, in the corotational formulation, where the material is taken stationary and thus **n** delivers no time rate, one simply has

(23) $$\dot{C}={\dot{n}}^{\left(3\right)}.n$$

The component of ${\dot{n}}^{\left(3\right)}$ along **m** can be derived considering that $n^{\left(3\right)}$ is a unit vector, which entails

(24) $${\left(n^{\left(3\right)}.m\right)}^{2}+{\left(n^{\left(3\right)}.p\right)}^{2}+{\left(n^{\left(3\right)}.n\right)}^{2}=1\Rightarrow {\dot{n}}^{\left(3\right)}.m=-\frac{C}{S}{\dot{n}}^{\left(3\right)}.n$$

Note that component $n^{\left(3\right)}.p$ does not deliver a time rate since no shear is exerted along **p**. Then, the identity ${\dot{n}}^{\left(3\right)}=\Omega^{l}n^{\left(3\right)}$ requires that $\Omega^{l}$ be expressed in the following format:

(25) $$\Omega^{l}=\frac{{\dot{n}}^{\left(3\right)}.m}{S}m⊗m+\frac{{\dot{n}}^{\left(3\right)}.n}{C}n⊗n$$

which, along with (20), leads to the following equivalent form:

(26) $$\Omega^{l}=\left(\frac{\dot{c}}{c}-\frac{1}{3}\left[\frac{\dot{f}}{f}+2\left(\frac{\dot{w}}{w}-\frac{\dot{\lambda }}{\lambda }\right)\right]\right)\left(n⊗n-{\left(\frac{C}{S}\right)}^{2}m⊗m\right)$$

where all terms and rates have been defined. Note that in the case of simple shear, as in [1], this equation simplifies considerably since

$$\dot{c}=0,\;\;\dot{f}=0,\;\;\dot{\lambda }=0$$

In summary, evolution Eqs. (16) and (17) with due account of (26) are those labeled (5), (6) and (7) in Ref. [1]. They were here derived for a rotating cylindrical void whose motion is constrained by the elastically unloaded zones above and below it. In [1], we heuristically use them for spheroidal voids. Similar equations can also be developed for rotating spheroidal voids. However, the geometry leads to more complex equations that would take away from the simplicity of the present treatment. Furthermore, when dealing with the special case of initially spherical voids ($w_{0}=1$) the void axis is arbitrarily defined. Under such circumstances, the principal stretch is used to resolve any indeterminacy upon deformation, as explained elsewhere [3]. In order to avoid this unnecessary complication, in [1] we have used a void with a slight initial eccentricity ($w_{0}=1.1$) so that its axis $n^{\left(3\right)}$ is well defined from the outset.

### Void closure

**Fig. 3:**
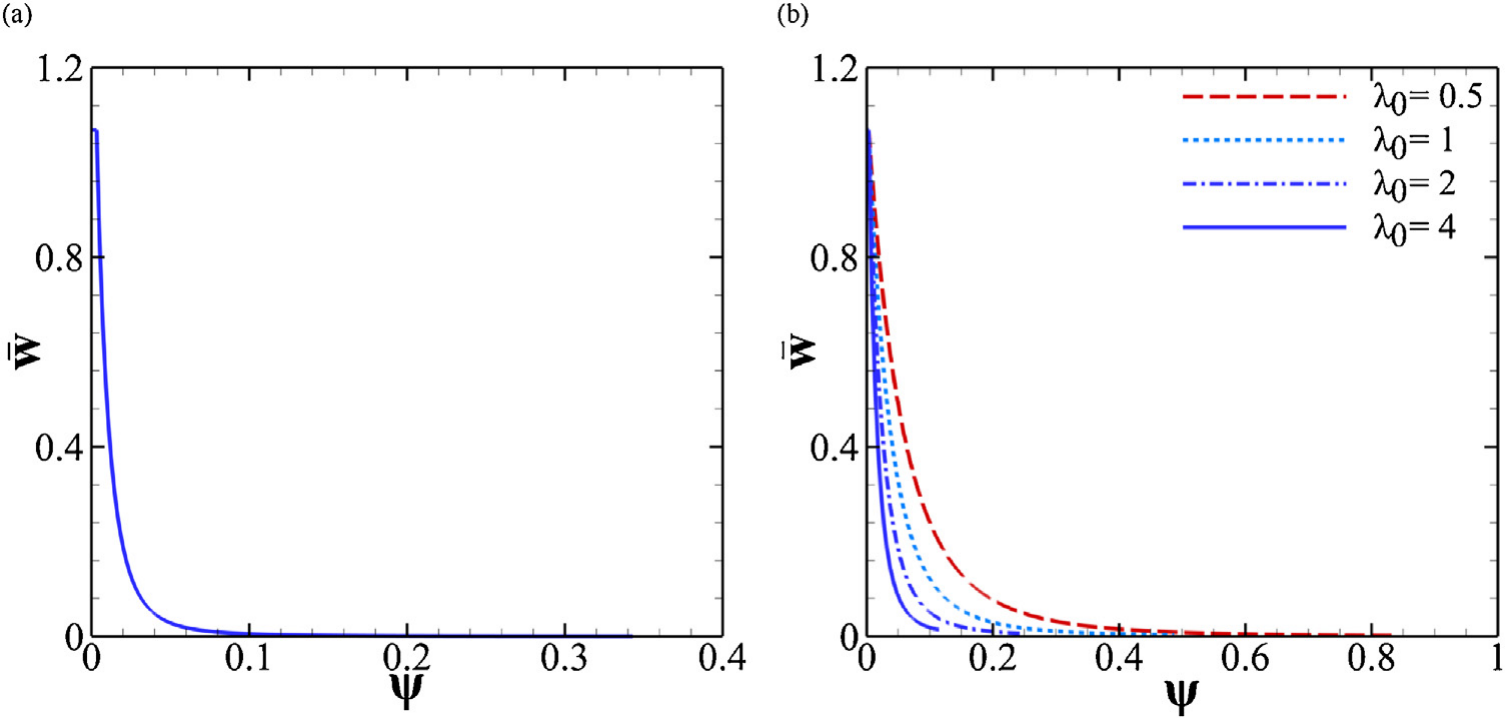
Void flattening in the surrogate microstructure by evolution of the effective void aspect ratio,$\bar{w}$ in (a) the case shown in Fig. 4 of Ref. [1] using $\sigma /\tau =0.01,\;\;f_{0}=0.0005,\;\;w_{0}=1.1,\;\;\lambda_{0}=2,\;\;N=0.2,\;\;\sqrt{3}\tau_{0}/E=0.002$; and (b) the cases shown in Fig. 6 of Ref. [1] using.

In exact finite element simulations of void growth in a shear field [[18], [19], [20]], the void volume fraction may slowly decrease if the tension-to-shear ratio is sufficiently low. By way of consequence, the void closes into a crack. Various ways of avoiding details pertaining to handling contact were explored by Tvergaard [[18], [19], [20]]. In our simulations, the void volume fraction *f* remains constant. As mentioned in [1], this is an approximation with no consequence on essential behavior, because *f* is not an essential internal state variable in simple shear. Since *f* is predicted to remain constant, void closure never occurs per se. However, it does in an asymptotic sense. To illustrate this, Fig. 3 shows the evolution of the effective void aspect ratio, $\bar{w}$, introduced in Eq. (5) for various cases reported in [1]. As the void rotates, its projected length onto the localization plane increases. Thus, to maintain equality of volumes between the actual void and the surrogate void, the latter must go increasingly flat, i.e. $\bar{w}\to 0$. This is illustrated in the surrogate microstructure of Fig. 1. The faster the rotation the more severe the flattening in the surrogate cell.

### Conclusion

A theoretical framework was developed for post-localized plastic flow in porous materials under shear-dominated loading. The constitutive relations account for the evolution of effective internal parameters defined via a surrogate microstructure emanating from the main one through a mere replacement of the rotated void and cell with equivalent upright counterparts. The latter operation is essential to describing the gradual loss of the shear load bearing capacity in that the conventional damage parameter denoting void volume fraction remains constant according to its evolution law under shear. A simple criterion for void closure due to void rotation and elongation in shear was also introduced and quantified in terms of the surrogate void aspect ratio. The major contribution of the proposed framework is to mimic the *de facto* failure mechanism under shear-dominated loading conditions.

## Footnotes

1 In [1], λ physically represents the ratio of shear band thickness to the in-plane void spacing. Void and cell are assumed to admit an axis of symmetry so that a one-parameter representation of their shapes is possible.
